# Current Perspectives in Antiviral Research

**DOI:** 10.3390/ijms241914555

**Published:** 2023-09-26

**Authors:** Olga A. Tarasova

**Affiliations:** Laboratory of Structure-Function Based Drug Design, Institute of Biomedical Chemistry, 10 Bldg. 8, Pogodinskaya Str., 119121 Moscow, Russia; olga.a.tarasova@gmail.com; Tel.: +7-92-6722-1890

## Editorial

Studies on virus–host interactions are of high significance for a number of reasons. First, comparing the molecular mechanisms of virus–host interactions allows researchers to reveal the complex relationships between specific viral infections and other pathologies that may develop after a particular viral infection and be associated with it. Meanwhile, for some viruses, their role in the development of cancer is shown and widely discussed, and some new potential associations between viral infections and tumor development are under active investigation. Specifically, for viruses such as Human Papilloma Viruses (HPV), their impact in the development of cervical cancer has been demonstrated and discussed [1]. The role of hepatitis B and C viruses in hepatocyte inflammation followed by necrosis and regeneration, associated with the fibrotic remodeling of hepatic tissue, leading to hepatocellular carcinoma has also been revealed [2]. Human immunodeficiency virus (HIV) infection is often associated with Hodgkin lymphoma [3,4], lung cancer [5], skin cancer [6], and Kaposi sarcoma [7]. An increased risk of gastric [8] and esophageal [9] cancers, and Hodgkin lymphoma [10,11] has been found to be associated with an Epstein–Barr virus-positive status. Potential associations between viruses and several tumor types are currently under investigation [12,13]. The role of different viruses in skin cancer is of interest to researchers and is under active study [12]. In a publication by Sara Becerril and co-authors, papillomaviruses responsible for the development of different skin tumors are discussed [12]. The impacts of several viruses, including influenza A, respiratory syncytial viruses, rhinoviruses, and some others on asthma development are discussed in the publication by Tuomas Jartti et al. [13]. The investigation of signaling pathways common to different pathologies can shed light on the relationships between virus–host interactions and the potential development of pathologies which develop as a consequence of viral infections, even when there are no known associations between them. For example, a study by Suguru Kadomoto and colleagues [14] demonstrates the involvement of chemokine receptors in viral infections and tumor pathogenesis. Additionally, Sidney Iriana et al. [15] reviewed the role of the Hedgehog signaling pathway in both cancer and viral infections. Another approach is to analyze the influence of virus-induced immune damage on the potential development of non-viral pathology. While the influence of HIV and SARS-CoV-2 on immune function continues to attract researchers’ attention [16,17], other viruses, such as herpesviruses, hepatitis C, influenza A, lymphocytic choriomeningitis virus and some others, have been studied for several years in terms of their impact on the immune response to infections [18,19,20,21,22]. An important field of research includes studies aimed at modeling the dynamics of viral copy spread in the host’s body [23].

Second, an analysis of virus–host interactions provides a basis for the search for new drugs with a pathogenesis-directed mechanism of action and symptomatic treatment. This makes it possible to alleviate symptoms in the severe course of viral infections and increase the clinical effectiveness of treatment. A thorough investigation of virus–host interactions for various viruses will enable researchers to increase the efficacy and decrease the potential time and costs of searching for novel medications for upcoming threats associated with the spread of zoonotic viral infections among the human population.

An estimation of the contribution of individual patient factors to the response of the immune system and other organs and tissues (nervous system, liver, kidneys, gut, endocrine system, etc.) at the genomic, transcriptomic, and proteomic levels will make it possible to predict the potential host’s response to viral infection and antiviral therapy due to a complex of factors. Additionally, such an analysis allows a determination of the possible level of progression of viral infection according to a set of individual characteristics of the patient [24], and a prediction of the development of complications and associated comorbidities that worsen quality of life [20,25,26,27].

Experimental knowledge about proteins and genes involved in the host response to viral infection provides a basis for predicting viral tropism, pathogenicity levels, and the ability of viruses capable of infecting animal tissues to also infect human organs and tissues. This knowledge also assists in the estimation of the risks associated with the spread of a specific virus within the human population.

An important area of current antiviral research is the search for new drugs, including analogs of known viral enzyme inhibitors with lower toxicity and susceptibility to the development of resistance [28,29,30,31]. The application of in silico methods allows for time and cost reductions, and the filtering out of potentially toxic chemical compounds, decreasing the probability of side effects for novel drugs. On the other hand, the application of in silico methods requires the production and collection of a lot of experimental data. The accumulated experimental data on antiviral activity available in scientific publications and large repositories has led to the development of new methods aimed at information extraction, collection, integration, and processing [32,33]. An analysis of data consistency and reproducibility would help to improve the accuracy of the models and the creation of benchmark data sets, which could be used by researchers worldwide for the validation of computational methods.

The prediction of viral drug resistance is highly significant and relevant for clinical practice, as it helps to optimize and increase the efficacy of antiviral therapy in a short time, improving patient prognosis. While there is great demand for studies analyzing and predicting, HIV drug resistance [34,35], investigations of the susceptibility of SARS-CoV-2 variants to pharmaceuticals [36] and their response to vaccines [37] are of high significance due to the pandemic.

Since solving all of the aforementioned issues depends on high-quality experimental data, it is important to consolidate the efforts of the international scientific community to create, develop, and annotate/curate freely available repositories, and maintain previously developed ones containing experimental data [38,39,40]. These data may encompass information on the interaction of the virus with the human body, the antiviral activity of low-molecular-weight and macromolecule compounds, the drug resistance of viruses, etc. An analysis of the negative results of experiments (failure to accept the initial hypothesis regarding the interaction between viruses and the human body, or the low affinity of a chemical compound against a viral target) could be of particular interest for researchers in related fields.

The development and evaluation of models aimed at understanding how viruses spread in the human body and virus–host interactions are beneficial for further antiviral drug research, including in the development of novel pharmaceutical agents, drug repositioning, as well as for the prevention of new threats associated with infectious diseases.
